# Exploring the dynamic interplay between exosomes and the immune tumor microenvironment: implications for breast cancer progression and therapeutic strategies

**DOI:** 10.1186/s13058-024-01810-z

**Published:** 2024-03-29

**Authors:** Sahar Safaei, Manouchehr Fadaee, Omid Rahbar Farzam, Amirhossein Yari, Elham Poursaei, Cynthia Aslan, Sahar Samemaleki, Dariush Shanehbandi, Behzad Baradaran, Tohid Kazemi

**Affiliations:** 1 Immunology Research Center, Tabriz University of Medical Sciences, Gholghasht Ave, Tabriz, Iran; 2 Department of Biology, Tabriz Branch, Islamic Azad University, Tabriz, Iran; 3 Biotechnology Research Center, Tabriz University of Medical Sciences, Tabriz, Iran; 4 Research Center for Integrative Medicine in Aging, Tabriz University of Medical Sciences, Tabriz, Iran; 5 Department of Immunology, Tabriz University of Medical Sciences, Tabriz, Iran

**Keywords:** Breast cancer, Tumor-derived exosomes, Immune cell, Tumor microenvironment, Immune cell-derived exosomes, Immunotherapy

## Abstract

Breast cancer continues to pose a substantial worldwide health concern, demanding a thorough comprehension of the complex interaction between cancerous cells and the immune system. Recent studies have shown the significant function of exosomes in facilitating intercellular communication and their participation in the advancement of cancer. Tumor-derived exosomes have been identified as significant regulators in the context of breast cancer, playing a crucial role in modulating immune cell activity and contributing to the advancement of the illness. This study aims to investigate the many effects of tumor-derived exosomes on immune cells in the setting of breast cancer. Specifically, we will examine their role in influencing immune cell polarization, facilitating immunological evasion, and modifying the tumor microenvironment. Furthermore, we explore the nascent domain of exosomes produced from immune cells and their prospective involvement in the prevention of breast cancer. This paper focuses on new research that emphasizes the immunomodulatory characteristics of exosomes produced from immune cells. It also explores the possibility of these exosomes as therapeutic agents or biomarkers for the early identification and prevention of breast cancer. The exploration of the reciprocal connections between exosomes formed from tumors and immune cells, together with the rising significance of exosomes derived from immune cells, presents a potential avenue for the advancement of novel approaches in the field of breast cancer therapy and prevention.

## Introduction

Breast cancer (BC) is the most common cancer among women in most countries, with the highest mortality and morbidity rates [1]. Despite the earlier detection and improved clinical management, there is still a high death rate among women because of treatment failure and metastasis [2]. BC can be divided based on its molecular characteristics into luminal A, luminal B, HER2, estrogen, and progesterone-positive receptors, and triple-negative BC (TNBC). Further subcategories of TNBC include basal-like 1 and 2, immunomodulatory, luminal androgen receptor, mesenchymal, and mesenchymal stem cell-like [3, 4]. TNBC and HER2-positive breast cancers are more likely to metastasize to other areas of the body, such as the brain, bone, lung, liver, and abdominal cavity [5, 6].

In recent decades, there has been a dramatic increase in the literature associated with the involvement of the immune system in BC [7]. It is well known that the immune system plays an important role in the development, progression, and control of BC [8]. The complex relationship between the immune system and cancer cells is characterized by the theory of “cancer immunoediting” (CI) [9], which was initially developed by Dunn et al. [10] CI postulates a dual role of the immune system in cancer: host-protective and tumor-promoting actions [11]. CI consists of “three Es”: elimination, equilibrium, and escape [12, 13]. In the ‘elimination’ phase, all tumor cells may be destroyed by the immune system [13]. During the second phase, the opposing forces remain balanced and cancer growth is still under control. Escape is the terminal stage of immunoediting, in which cancer cell variants effectively evade immune pressure, replicate progressively, and become clinically apparent [13]. In light of this, manipulating the immune system and boosting its defenses against cancer may be one of the keys to controlling cancer [14]. This idea is known as cancer immunotherapy [14]. Exosomes (Exo) have emerged as potential sources of biomarkers for early detection and prognosis in breast cancer. Several studies have identified specific molecules, such as proteins, nucleic acids, and microRNAs, within exosomes that show promise as diagnostic and prognostic markers. MiRNAs are small non-coding RNA molecules that regulate gene expression. For example, miRNA-21, miRNA-155, and miRNA-210 have been found to be upregulated in breast cancer-derived exosomes and are associated with tumor growth, metastasis, and poor prognosis [15–17]. In addition, Proteins such as human epidermal growth factor receptor 2 (HER2), and mucin 1 (MUC1) have been detected in exosomes isolated from breast cancer patients. Changes in the levels of these proteins in exosomes have been associated with tumor progression and can potentially serve as diagnostic and prognostic markers [18, 19]. The field of exosome research is rapidly evolving, and ongoing studies continue to identify new potential markers. The most common immunotherapy approaches in BC are non-specific immune stimulation, immune checkpoint blockade, adoptive cell transfer, and vaccination [20, 21]. Immune checkpoint inhibitors targeting programmed cell death 1 (PD-1), programmed cell death 1 ligand 1 (PD-L1), and cytotoxic T-lymphocyte-associated protein 4 (CTLA-4) have revolutionized cancer treatment by eliciting durable objective responses in multiple cancer types, often leading to improved overall survival [22, 23]. According to accumulating evidence, PD-1/PD-L1 inhibitors have the potential to induce durable clinical responses in some patients with metastatic TNBC and are likely to have clinically meaningful results in rare ER + HER2-BC patients as well [11]. Recent evidence has confirmed exosome-based immunotherapy as a potential new approach in the treatment of BC [24–26]. Exosomes are nano-sized small extracellular vesicles with endogenous origin and capacity to transport various types of proteins, lipids, RNA, microRNA (miR), and DNAs [27, 28]. Exosomes protect their cargo contents from degradation (especially RNA molecules) in systemic circulation [29]. In addition, their small size enables them to overcome the blood–brain barrier and penetrate deep into the tissues [29, 30].

Exosomes may serve as a robust and feasible vehicle for delivering immune-modulatory agents into specific target cells to either stimulate antitumor immunity or exert immunosuppressive effects [31]. In addition, Exosomes can deliver factors involved in DNA repair pathways, enhancing the repair of DNA double-strand breaks and promoting cell survival and radioresistance [32]. They can also transfer anti-apoptotic proteins that protect cancer cells from radiation-induced cell death [33]. Exosomes secreted from HER2-positive cell lines, SKBR3 and BT-474, are reportedly enriched in antigen processing/presentation-related proteins, including HSPA5 (heat shock 70 kDa protein 5), CALR (calreticulin), and PSME2 (proteasomeactivator complex subunit 2), and glycolysis/gluconeogenesis-related proteins, such as TPI1 (triosephosphate isomerase 1) and PGAM1(phosphoglycerate mutase 1) [34]. Interestingly, it has also been shown that SKBR3 and BT-474 derived-exosomes contain a full-length HER2 active molecule which can bound to trastuzumab and inhibit drug’s activity [35].

In this review, we aim to highlight the potential of using exosomes in the field of BC immunotherapy. We describe the known functions of BC-derived exosomes and immune cell-derived exosomes in immune modulation. Next, we point out how exosome-based immunotherapy can have significant therapeutic effects on BC progression. And finally, we will discuss the current knowledge on the possible applications of exosome-based immunotherapy in BC and the future direction of these fascinating vesicles in cancer therapy.

## Biogenesis of exosomes and their biological importance

Extracellular vesicles (EVs) are membrane-bound vesicles that are commonly classified into three main subtypes: exosomes, microvesicles (MVs), and apoptotic bodies [36]. For the first time, Johnstone et al. used the word “exosome” to describe small membrane vesicles formed in multivesicular bodies (MVBs) in the late 1980s [37]. Exosomes are generated by various cell types and loaded with a myriad of bioactive molecules, including nucleic acids, proteins, lipids, and other metabolites, which mediate cell–cell communication via their cargo contents [38]. They have a diameter of 30–150 nm, a typical “cup dish” shape, and a density of 1.13–1.19 g/mL [39].

The complex process that leads to the biogenesis of exosomes may be divided into three main steps: first, the early and late endosomes are formed; then, intraluminal vesicles (ILVs) are formed inside the late endosomes, which leads to the formation of MVBs; and finally, upon the fusion of MVBs with the plasma membrane, ILVs are secreted throughout body fluids (blood, saliva, urine, etc.) by exocytosis and are called exosomes [40, 41]. Exosomes can be released during normal and pathological states from a majority of cell types [42].

The process of cargo sorting into the ILVs is mediated via two opposing sorting systems namely, endosomal sorting complexes required for the transport (ESCRT) dependent and ESCRT-independent pathways [41]. Four ESCRT proteins (ESCRT-0, ESCRT-I, ESCRT-II, and ESCRT-III), tumor susceptibility gene 101 (TSG101), and ALIX are mainly involved in the transition of endosomes to exosomes [43, 44]. The ESCRT-I protein Tsg101, together with the ubiquitinated proteins, triggers activation of the ESCRT-II complex and mediates the oligomerization of the ESCRT-III complex [45]. ESCRT-III machinery contributes to vesicle release into the endosome and scission [40]. Vacuolar protein sorting associate protein 4 (VPS4) and ATPase degrade the ESCRT-III complex as the final step of exosome formation [40, 46]. However, under certain conditions, the biogenesis of exosomes can occur via an ESCRT-independent pathway. This process can be mediated by lipids such as ceramides and cholesterol [47].

**Fig. 1:**
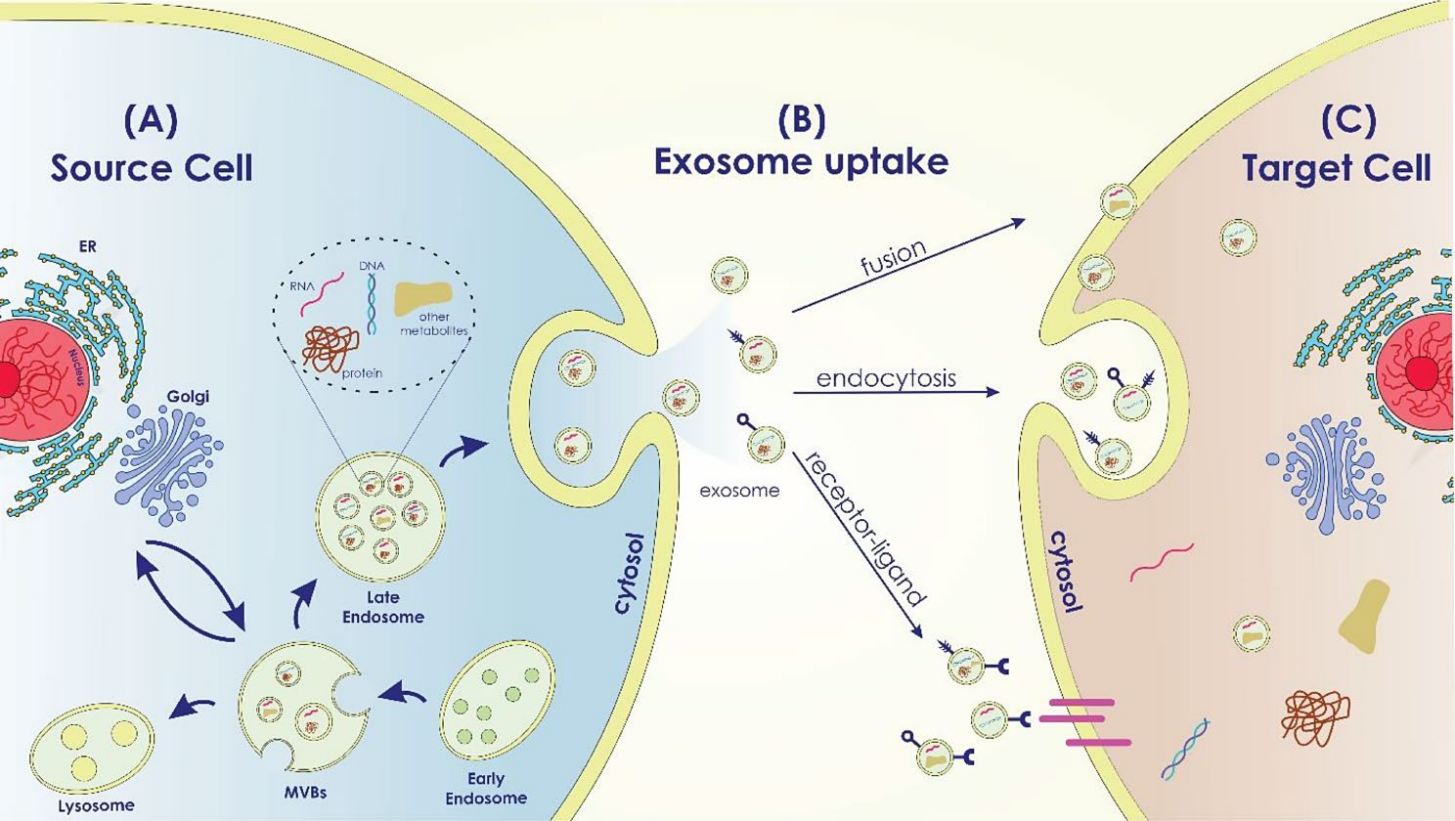
Exosome Biogenesis in the Source Cell, Uptake, and Cargo Transfer (**a**) Exosome Biogenesis in the Source Cell: The intracellular mechanisms involved in exosome biogenesis within the source cell are depicted in this area of the image. It displays the early endosome formation process, the sorting of different cargo molecules into the developing exosomes, and the inward budding of the endosomal membrane to generate multivesicular bodies (MVBs). Labeled cargo molecules consist of proteins, RNAs (miRNAs and mRNAs), lipids, and other bioactive compounds. (**b**) Exosome Uptake Routes by Target Cells: This portion of the diagram shows the various ways that target cells can absorb exosomes. It demonstrates a variety of exosome uptake processes, including as receptor-mediated interactions, membrane fusion, and endocytosis. (**c**) Cargo Transfer and Impacts in the Target Cell: The cargo transfer that occurs when exosomes enter the target cell and the ensuing impacts are the main topics of this portion of the picture. It shows how certain cellular processes are activated and how exosome cargo is released into the target cell’s cytoplasm

Exosomes participate in several normal and pathological conditions, such as pregnancy, immune responses, cardiovascular disorders, viral pathogenesis, neurological disorders, and cancer [48]. According to the origin of the donor cell, exosomes play a variety of roles, including immune response, antigen presentation, programmed cell death, angiogenesis, inflammation, coagulation, etc. [27, 49] (Figure1).

## TDEs effect on immune cells

**Fig. 2:**
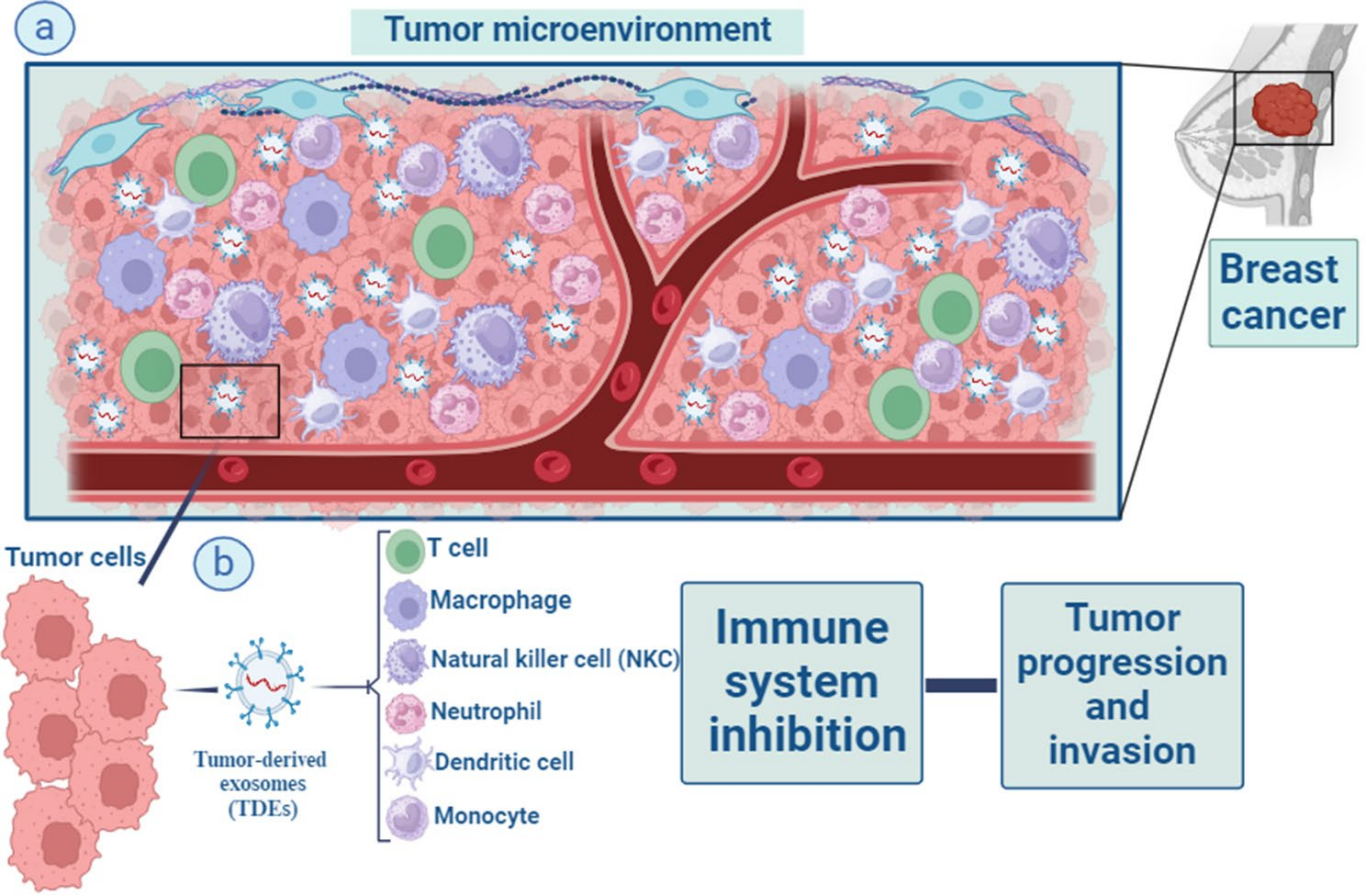
Macrophages, T lymphocytes, natural killer (NK) cells, dendritic cells, neutrophils, and monocytes are all part of the intricate web of immune cells that surrounds tumour and impacts cancer development and outcome via their interactions with tumour cells. Important immune modifiers in the tumor environment are something called tumor-derived exosomes (TDEs) (**a**). TDEs enhance the development of tumors by lowering and suppressing the level of immune cells cytotoxicity and activities in the microenvironment around tumor cells. They do this by transferring suppressive cargo to these cells (**b**)

Over the past 10 years, the study of exosomes has observed increasing attention, representing a high-potential area for their application in cancer as biomarkers and therapeutic tools [50]. Tumor-derived exosomes (TDEs) are important immune adjusters in the tumor milieu. TDEs reduce immune cell cytotoxicity in the microenvironment of tumor cells by transporting suppressive cargo to these cells, to facilitate tumorigenesis [51]. TDEs have been implicated in all stages of BC progression (tumor initiation, growth, dissemination, and metastatic spread) [2]. As part of their immunological functions, exosomes modulate antigen presentation, immune activation, immune surveillance, intercellular communication, and immune suppression [52, 53]. On the other hand, the expression of PD-1 on the surface of BC-derived exosomes can blunt T-cell activation, effectively enabling tumor cells to escape immune surveillance [54]. The functions of exosomes secreted from cancer cells are discussed in more detail in the following sections (Fig. 2).

### NK cells

NK cells, a subset of innate effector lymphocytes, need constant renewal through bone marrow cells. It is well known that Natural killer (NK) cells are highly effective in attacking tumor cells. NK cells directly eradicate tumors using death receptors and cytolytic particles. There is evidence that Fas ligand (FasL) expressed on the surface of NK cell-derived exosomes is involved in killing Fas + tumor cells [55]. They defend against metastatic and initial tumor cells, and enhance adaptive immunity by releasing cytokines to inhibit target cell growth [56].

TDEs can integrate with the NK cell membrane, obstructing NK cells’ antitumor activity by carrying their cargo to NK cells [57, 58]. In addition, the interplay among receptors on NK cells and their TDE ligands excites multiple downstream inhibitory signaling pathways that suppress NK cells’ cytotoxic function [51]. Examples of NK cells activating receptors that TDEs downregulate include NKp46, NKG2C, NKp30, and NKG2D [59]. The exosomes-derived breast carcinoma cell line T47d, even in the presence of IL-15 stimulation, inhibits immunological functions via reducing NKG2D receptor expression [60]. The ratio of CD56^dim^ and CD56^bright^ NK cells in patients with breast cancer in relation to negative control was remarkably increased and decreased, respectively [61].

**Fig. 3:**
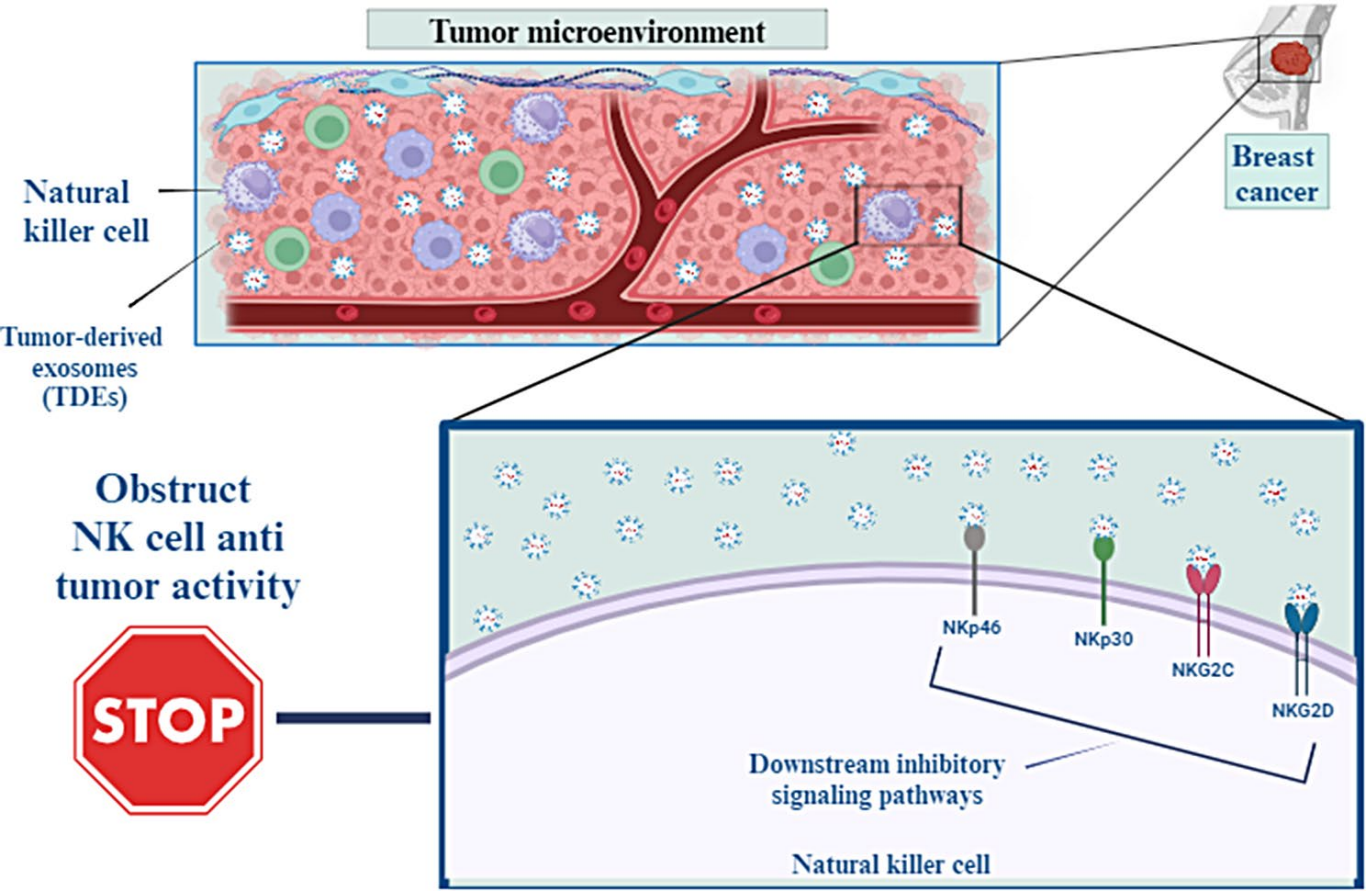
TDEs can integrate with the membrane of NK cells, which may inhibit the antitumor activity of NK cells by transporting their payload to these cells. Furthermore, the interaction between receptors on NK cells and the TDE ligands that bind to them activates many downstream inhibitory signaling pathways, which in turn reduce the cytotoxic activity of NK cells

Stimulating NK cells by releasing (IFN-γ) and (TNF-α) promote the antitumor immune reaction. However, purified exosomes from mouse breast cancer cells debilitated the production of IFN-γ from IL-2– activated NK cells and, so impaired NK cell proliferative capacities. TDEs also significantly inhibited human NK cell proliferation in response to IL-2 through inhibiting Janus kinase 3(Jak3)-mediated signal transducers and activators of transcription 5(STAT 5) stimulation. Moreover, TDEs intensely decreased perforin release in IL-2 motivated NK cells [62]. Wen et al. demonstrated that breast cancer–derived exosomes reduced NK cells’ number and anticancer abilities [63] (Fig. 3).

### T-CD8 + cells

A subpopulation of T cells known as T-CD8 + cells play a crucial role in the immunological tumor microenvironment of cancer patients because of their capacity for cytotoxicity. Through the use of major histocompatibility complex class I (MHC-I) molecules and the T cell receptor (TCR) found on T-CD8 + cells, they are able to identify tumor-associated antigens expressed on the surface of cancer cells. Tumor progression, invasion, and resistance to therapies are closely related to tumor microenvironment (TME) interactions. Cancer cells can evade antitumor immunity through packaging immunosuppressive mediators, such as FasL, TNF-associated apoptosis inducing ligand (TRAIL), transforming growth factor-β (TGF-β), and PD-L1 into their exosomes and affect T-CD8 + cell activity [64].

**Fig. 4:**
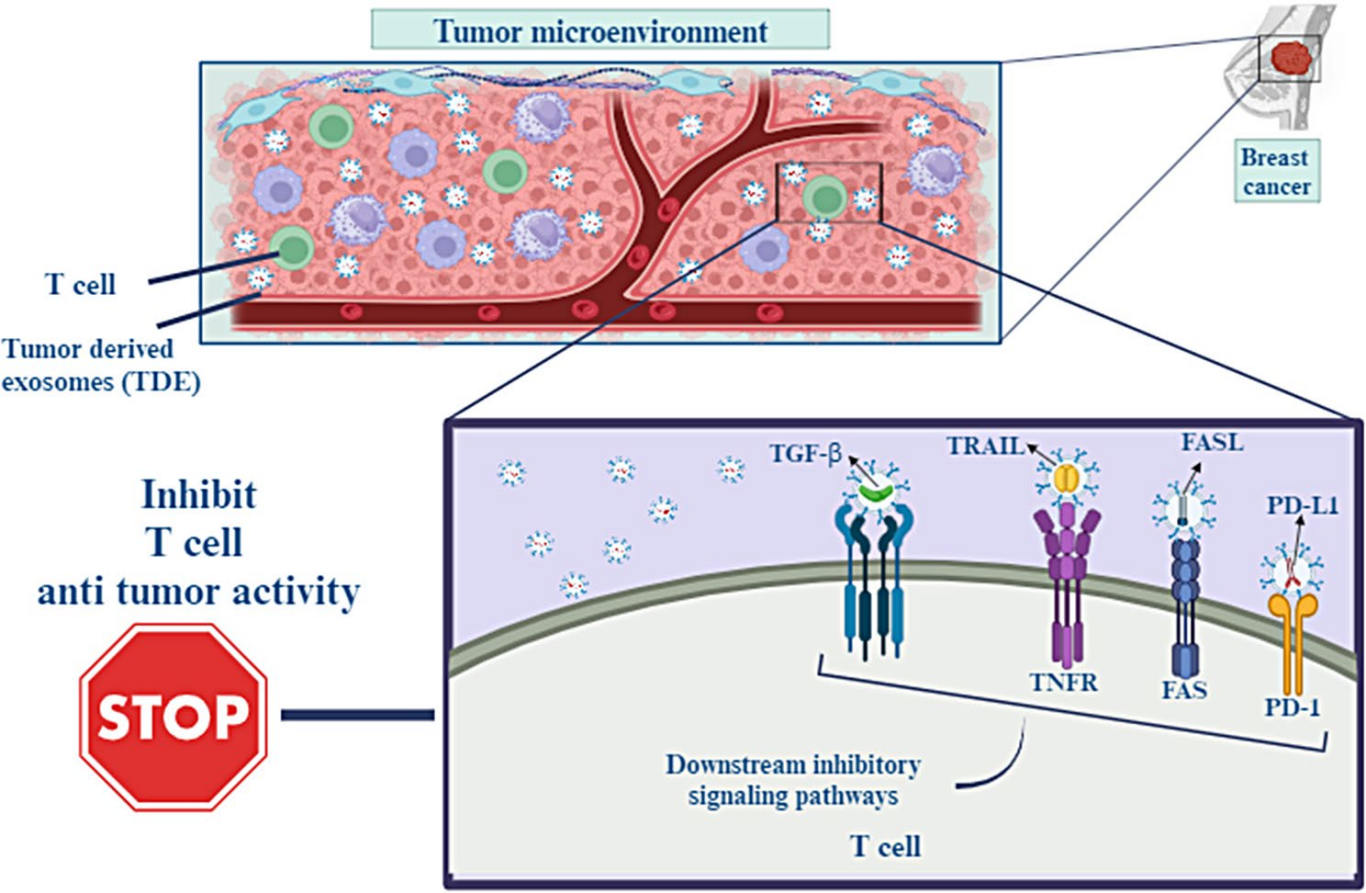
Interactions within the tumor microenvironment (TME) are directly connected to the growth of cancer, its ability to invade surrounding tissue, and resistance to treatment. Cancer cells can resist antitumor immunity by packing immunosuppressive mediators including FasL, TNF-associated apoptosis-inducing ligand (TRAIL), transforming growth factor- β (TGF- β), and PD-L1 into their exosomes. This allows the cancer cells to circumvent the immune system

A high level of PD-L1 expression is associated with a poor prognosis in various types of tumors. PD-L1 binds to the PD-1 receptor which is expressed on almost all immune cells and inactivates their function. So, immune checkpoint inhibitors target the PD1/PD-L1 axis in cancer immunotherapy. However, anti-PD-L1 antibodies are inefficient at blocking exosomal PD-L1, so this phenomenon can promote immunotherapy resistance [65]. In head and neck squamous cell cancers (HNSCC) patients, the level of PD-L1 on exosomes, but not the level of soluble PD-L1, correlates with the progression of the disease and poor prognosis [66]. In a study, TNBC exosomes overexpressing PD-L1 reduced T-CD8 + numbers and inhibited their activities, whereas the differentiation of pro-tumorigenic cells including myeloid-derived suppressor cells (MDSCs) and M2 macrophages increased in TME [67]. T-CD8 + granzyme B secretion is also inhibited by exosomal PD-L1 in vivo, thereby protecting and promoting tumor growth [54]. Chatterjee et al. showed that high level of transforming growth factor beta (TGF-β) is correlated with secretion of PD-L1 carrying exosomes from BC cells [68]. Synergistic effects of TGF-β and TDEs expressing high PD-L1 impair T-CD8 + function by suppressing T cell receptor (TCR) signaling mediated phosphorylation (Fig. 4).

### Tregs

Regulatory T cells (Tregs) assist tumor cells in evading immune system responses by secreting immunosuppressive cytokines including IL-10 and TGF-β1. The presence of Tregs within tumors is closely linked to cancer prognosis [69]. Studies have shown that exosomes obtained from tumor cells promote the production and proliferation of Tregs. CD25^−^ CD4^+^ T cells can be transformed into FoxP3^+^ CD4^+^ CD25^high^ T cells via coculture with exosomes derived from tumors. Exosomal microRNA-214, in terms of its mechanism, suppresses the expression of phosphatase and tensin homolog (PTEN) in T cells and induces regulatory T cells (Tregs) to secrete IL-10, which ultimately leads to the promotion of tumor growth [70]. Exosomes of mutant KRAS lung tumor cells promote the conversion of naïve T cells into Tregs, a phenomenon also seen following the cDNA transfection of mutant KRAS. DNA of exosomal mutant KRAS causes this transformation, as evidenced by the increased presence of FoxP3 + Tregs in cancer tissues of mutant KRAS patients compared to those with wild type (WT) KRAS [71]. Moreover, in breast cancer, exosomal long noncoding RNA (lncRNA) SNHG16 has been shown to promote the development of CD73 + γδ1 Treg cells in breast cancer by sequestering miR-16-4p, leading to the suppression of SMAD5, activation of the TGF-b1/SMAD5 pathway, and ultimately increasing CD73 expression [72].

### MDSCs

Myeloid-derived suppressor cells (MDSCs) are immature myeloid-derived cells with various morphologies present in the tumor microenvironment, possessing potent immune suppressive capabilities [73]. The aggregation of MDSCs inhibits antigen processing and it’s presentation and releases several immunosuppressive substances that disrupt immune responses [74]. Several studies have indicated that the suppressive function of MDSCs on T cells can be potentiated by cancer exosomes. Exosomal HSP70 in renal carcinoma promotes cell growth and boosts the activity of MDSCs by activating TLR2 signaling [75]. Meanwile, miR-181a and miR-9 and from exosomes produced from breast cancer promote tumor development and immune escape by boosting the activity of MDSCs through activating the JAK/STAT signaling pathway. This is achieved by individually suppressing SOCS3 and PIAS3 [76]. In addition, Exosomes derived from breast cancer contain TGF-β1 and PGE2, which promote the aggregation of MDSCs and facilitate tumor progression [77].

### Non-immune cells

Exosomes play a significant role in modulating the tumor microenvironment by interacting with various stromal cell types, including cancer-associated fibroblasts (CAFs), endothelial cells, and adipocytes [78]. BC-Exos can educate and activate CAFs, leading to the secretion of growth factors, cytokines, and extracellular matrix components that promote tumor growth and invasion. Moreover, exosomes can transfer oncogenic signals, such as transforming growth factor-beta (TGF-β), to CAFs, further promoting tumor progression and epithelial-mesenchymal transition (EMT) [79]. In addition, BC-Exos stimulate angiogenesis by promoting endothelial cell migration, proliferation, and tube formation. These exosomes carry pro-angiogenic factors, including vascular endothelial growth factor (VEGF), fibroblast growth factor (FGF), and matrix metalloproteinases (MMPs), which enhance the formation of new blood vessels to support tumor growth and metastasis [80, 81].

In summary, exosomes have the ability to influence the composition and functions of the tumor microenvironment. By inducing phenotypic changes in cancer-associated fibroblasts and immune cells, they promote the secretion of factors that contribute to radioresistance or immunosuppression. This altered microenvironment provides a supportive niche for surviving cancer cells and contributes to treatment resistance [82].

## Exosomes-based immunotherapy approaches for BC

Surgical resection, chemotherapy, radiotherapy, hormone therapy, and radiation therapy are currently available to breast cancer patients for treatment. However, drug resistance and toxicity to healthy tissues are two issues that plague all current treatments [83]. So, developing targeted, non-toxic, and non-immunogenic delivery technologies is crucial. Exosomes and the tumor microenvironment are becoming increasingly attractive targets for clinical applications. This is mainly because of their adaptable function in carcinogenesis in terms of cancer diagnosis and response to treatment [84].

Precision BC treatment has been significantly improved by nanoparticle-based technology. Exosomes are natural nanoparticles carrying chemotherapeutic drugs, photosensitizers or antitumor drugs. For cancer therapy, exosomes are particularly appealing drug delivery system due to their excellent biosafety, low immunogenicity, carrier properties, and nanoscale penetration effect [85] (Table 1). The exosomes primary advantage over other nanoparticles is inherent capacity to transport molecules of biological significance. This feature has prompted research in treatment choices drugs including the utilization of nucleic acid or enactment of the immune system.

**Table 1:** Overview of Exosome based immunotherapy approaches for BC

Citation	Exosomes-based immunotherapy	Population description	intervention	Exposure assessment	Brief result
(Huang, Rong et al. 2022) [86]	Exosomal vaccine	TNBC mouse, MDA-MB-231/Luc cells	α-LA is used to engineer exosomes derived from breast cancer cells. These exosomes are then combined with ELANE and Hiltonol to create a DC vaccine called HELA-Exos.	flow cytometry, Western blot	The HELA Exosomes can induce immunogenic cell death (ICD) in breast cancer cells in a targeted manner.
(Wang, Wang et al. 2019) [87]	Encapsulated drug	Balb/c mice, 4T1 cells and RAW264.7 cells	Exosomes derived from M1-macrophages were utilized as carriers for PTX.	Western blot, RT-PCR Analysis, ELISA assay, Caspase-3 activity assay, MTT (3-[4,5-dimethylthiazol-2-yl]-2,5 diphenyl tetrazolium bromide) Assay, Apoptosis assay	The M1-Exos serves as a carrier to deliver PTX to tumor tissues and improve the anti-tumor effects of chemotherapeutics in mice with tumors.
(Yu, Gai et al. 2019) [88]		MDA-MB-231 and HFL-1	Exosomes loaded with erastin and labeled with FA were vectorized to target TNBC cells (erastin@FA-exo).	Western blot analysis, Colony forming assay, Wound healing assay, Flow cytometric analysis	Erastin from folate-Exo can selectively target and induce ferroptosis in MDA-MB-231 tumor cells
(Zhao, Gu et al. 2020) [89]	SiRNA exosome	HUVEC cells and 4 T1 mouse breast cancer cells	CBSA conjugated siS100A4 and exosome membrane coated biomimetic nanoparticles (CBSA/siS100A4@Exosome) that consist of a CBSA/siS100A4 core and an exosome membrane shell	Western blot, Wound healing assay, Immunofluorescence	CBSA/siS100A4@Exosome-mediated RNAi was demonstrated to down-regulate the expression of S100A4, induced significant suppression of postoperative metastasis in triple-negative breast cancer
(Ohno, Takanashi et al. 2013) [90]	Exosome-mediated exogenous mRNA	RAG2–/– mice, HCC70, HCC1954, and MCF-7 cells, HEK293	Intravenously injected exosomes delivered let-7a miRNA to EGFR-expressing xenograft breast cancer tissue	Western blot, real-time reverse transcription-PCRs, Flow cytometry	Exosomes can target cancerous tissues expressing EGFR with nucleic acid drugs
(Gong, Tian et al. 2019) [87]		, THP-1 cells and B16 cells	The A15-Exo has been equipped with a chemotherapy drug and miRNA159 modified with cholesterol	Confocal microscopy, Apoptosis assay, Quantitative real-time PCR	A15-Exo loaded with a chemotherapeutic drug and cholesterol-modified miRNA159 were used for in vitro and in vivo experiments. The treatment induced a synergistic therapeutic effect in MDA-MB-231 cells and exhibited improved anticancer effects
(Han, Wang et al. 2020) [91]	NK derived exosome	NK-92 cells, MCF-7 cells	effectiveness of NK-Exos-entrapped PTX-NK-Exos	Western blotting, HPLC, MTT assay, flow cytometry, qRT-PCR	According to qRT-PCR and Western blotting, PTX-NK-Exos induced apoptosis in tumor cells by up-regulating Bax and Caspase-3
(Hashemi, Ghavami et al. 2023) [92]		breast cancer spheroids by NK cells	delivery of SFB to breast cancer spheroids by NK-Exos	MTT assay, RT-PCR, western blot, scratch, and migration assay, electroporation	Spheroids treated with SFB-NK-Exos exhibited increased cytotoxicity and apoptosis despite a reduced SFB concentration in the formulation, similar to that of free SFB

*Note* α-LA alpha-lactalbumin. ELANE human neutrophil elastase. Hiltonol TLR3 agonist. DC dendritic cell. 4 T1 mouse breast cancer cells. PTX Paclitaxel. MDA-MB-231 human TNBC cell line. HFL-1 human fetal lung fibroblasts. FA folate. HUVEC human umbilical vein endothelial cells. RAW264.7macrophage cells. CBSA cationic bovine serum albumin. HCC70, HCC1954, and MCF-7 Breast cancer cell lines. human monocytes HEK293 and THP-1 cells. B16 cells, BALB/C-nu mice. EGFR Epidermal growth factor receptor. NK-Exos natural killer cell-derived exosome. HPLC high-peperformanceiquid chromatography. qRT-PCR quantitative reverse transcription-polpolymeraseain reaction. SFB sorafenib

### Dendritic-based exosomes

Dendritic cells (DCs) play key role in the immune response by capturing, processing and presenting antigens (Ag) to immature T lymphocytes and activating them. This creates a crucial link between innate and adaptive immune responses. However, tumor-associated DC with immunosuppressive activity can induce T cell tolerance and progressive tumor growth. In order to take advantage of DC’s capacity to initiate and enhance anti-tumor immunity, novel DC-targeted therapies must be developed [93].

**Fig. 5:**
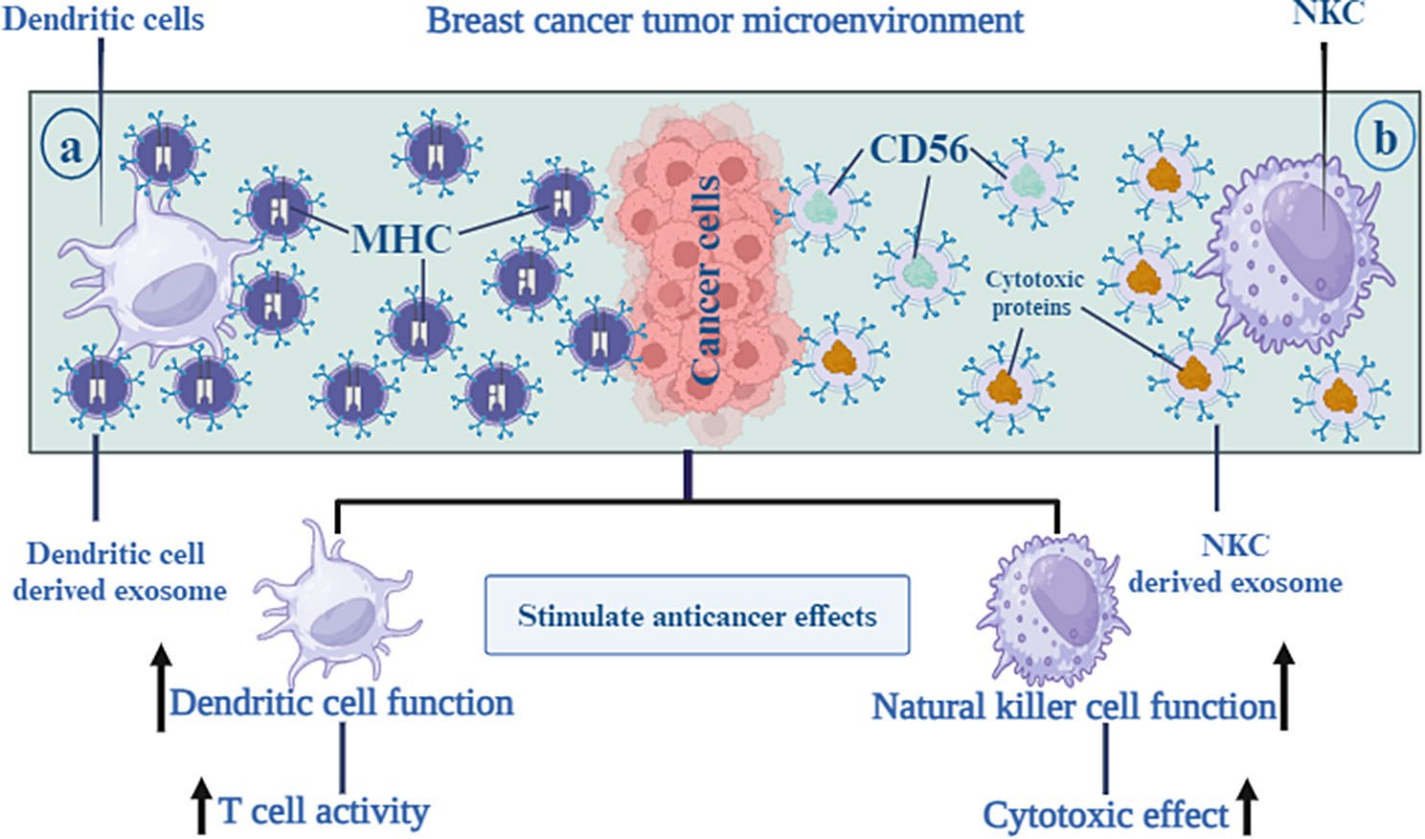
As an alternate delivery mechanism for tumor antigens, DC-derived exosomes, also known as Dex, have been attracting a lot of attention recently. Antigen-presenting particles like MHC-I and MHC-II, CD80 and CD86, and Hsp 70 and 90 are carried by dex in the body. This enables the DC to increase Tcell activity towards tumor tissue areas (**a**). NK-derived exosomes, also known as NK-Exos, are vesicles that include NK cells’ prototype markers, such as CD56, together with cytotoxic proteins like FasL and perforin. This enables the NK cells to guide their cytolytic activity towards tumor tissue areas (**b**)

In recent years, DC-derived exosomes (Dex) have been gaining interest as an alternative delivery method for tumor antigens. Dex carry antigen presenting particles such as MHC-I and MHC-II, CD80, CD86 and Hsp 70 and 90 [94]. T cells can be stimulated both directly and indirectly by Dex. In a direct manner, T cells are induced by MHC and co-stimulatory molecules on the Dex membrane [95]. However, this mechanism is not effective in activation of naïve T cells due to insufficiency of TCR-cross linking and co-stimulation [96]. As opposed to direct mechanisms, indirect mechanisms produce more potent T-cell responses against antigens since bystander DCs efficiently activate antigen-specific T cells in response to antigen substances transferred by Dex. Dex can induce T cell responses against the tumor by transferring MHC-peptide complexes to the tumor cell surface. One study showed that Dex treated human breast adenocarcinoma cells, SK-BR-3, increased IFN-γ secretion by CD3 + primed T cells [97] (Fig. 5a).

### NK derived exosome

In cancer immunotherapy, natural killer (NK) cells are being investigated for adoptive cell therapy as intrinsic lymphocytes that facilitate tumor immune surveillance [98]. It has also been demonstrated that NK cells control the immune response in other, non-cell activation-dependent ways, one of which is through exosomes [55]. NK derived exosomes (NK-Exos) containing NK cell’s prototype markers, e.g. CD56 and cytotoxic proteins such as FasL and perforin, enables them to direct their cytolytic activity into tumor tissue sites [99]. Zhu et al. developed exosome mimetics (NK-EM) by extruding NK cells through filters with increasingly smaller pores. NK-EM showed powerful tumor-killing activity against cancers such as glioblastoma and BC in mice as compared to NK-Exo at low and high doses [100]. Kaban and colleagues, transduced lentivirally NK cell line NK92MI to express and load BCL-2 siRNA (siBCL-2) into NK-Exos. siBCL-2 NK-Exos prompted intrinsic pathway of apoptosis in BC cells via targeting BCL-2, without influencing non-dangerous cells [101] (Fig. 5b).

### Exosome based vaccines

Cancer immunotherapy mainly relies on CD8 + T cells to promote Cytotoxic T-lymphocyte (CTL) activity, initiate tumor-specific CTLs in lymphoid organs, and establish lasting anti-cancer immunity [102]. Utilizing an EXO-based vaccine by activating the immune response is a promising cancer treatment option because the immune system avoids tumors. Wang et al. used HER2/neu-specific Neu-T cell derived exosome (TEXO) and HER2-TEXO vaccines by transfecting DCs to stimulate HER2/neu-specific CTLs and study anti-tumor activity. They demonstrated that novel T cell-based exosome vaccine was effective in inducing protective immunity against tumor cells and may be an alternative therapeutic method for HER2 + breast cancer, especially for women with trastuzumab-resistant HER2 + breast cancer [103]. Additionally, vaccination with exosome-loaded T cells (exosome-T) has the capacity to reverse CD4 + CD25 + Treg cell-mediated immunosuppression and initiate long-term CTL memory, making it an appealing method for generating immune responses against human cancers [104, 105]. Also, the HER2-specific exosome-T vaccine was just recently industrialized to effectively boost the immune system of the patient against HER2-positive breast cancer [106]. By utilizing the transmembrane domain of the human platelet-derived growth factor receptor to demonstration two distinct kinds of monoclonal antibodies on the external of an exosome, the brand-new synthetic poly valent antibody redirected exosome (SMART-EXO) was created. SMART-EXOs with the breast cancer-related HER2 and EGFR receptors can activate CTLs by targeting the CD3 receptor on the surface of T cells. CTLs then display highly effective and specific anti-tumor activity in vitro and in vivo [107, 108]. On the other hand, CAR-T exosomes have high safety and efficiency, which could enhance BC immunotherapy [109]. MSLN-positive TNBC cells and xenograft tumors growth were effectively inhibited by CAR-T cell exosomes secreting granzyme B and perforin without any overt side effects [110].

### Macrophage-based exosomes

Macrophages are one of the innate immune cells that play a critical role in TME. Macrophages have to major subtypes: M1 and M2. M1 macrophages have pro-inflammatory function and their polarization is due to several factors including Th1 cytokines, lipopolysaccharide (LPS), and Toll-like receptor (TLR) agonists. However, Th2 cytokines including IL-4 and IL-13, polarize macrophages to M2 phenotype that exert anti-inflammatory function and has involved in tissue repair and hemostasis [111]. Tumor-associated macrophages (TAMs) are one of the M2 subtypes that polarize from macrophages in the TME and facilitate tumor growth and angiogenesis. IL-6 signaling through signal transducer and activator of transcription 3 (STAT3) transcription factor phosphorylation, induce TAMs development and tumorigenesis. Ham et al. revealed that BC-derived exosomes render IL-6Rβ (gp130) to macrophages and stimulate STAT3 signaling, thereby enhancing pro-tumorigenic microenvironment [112].

**Fig. 6:**
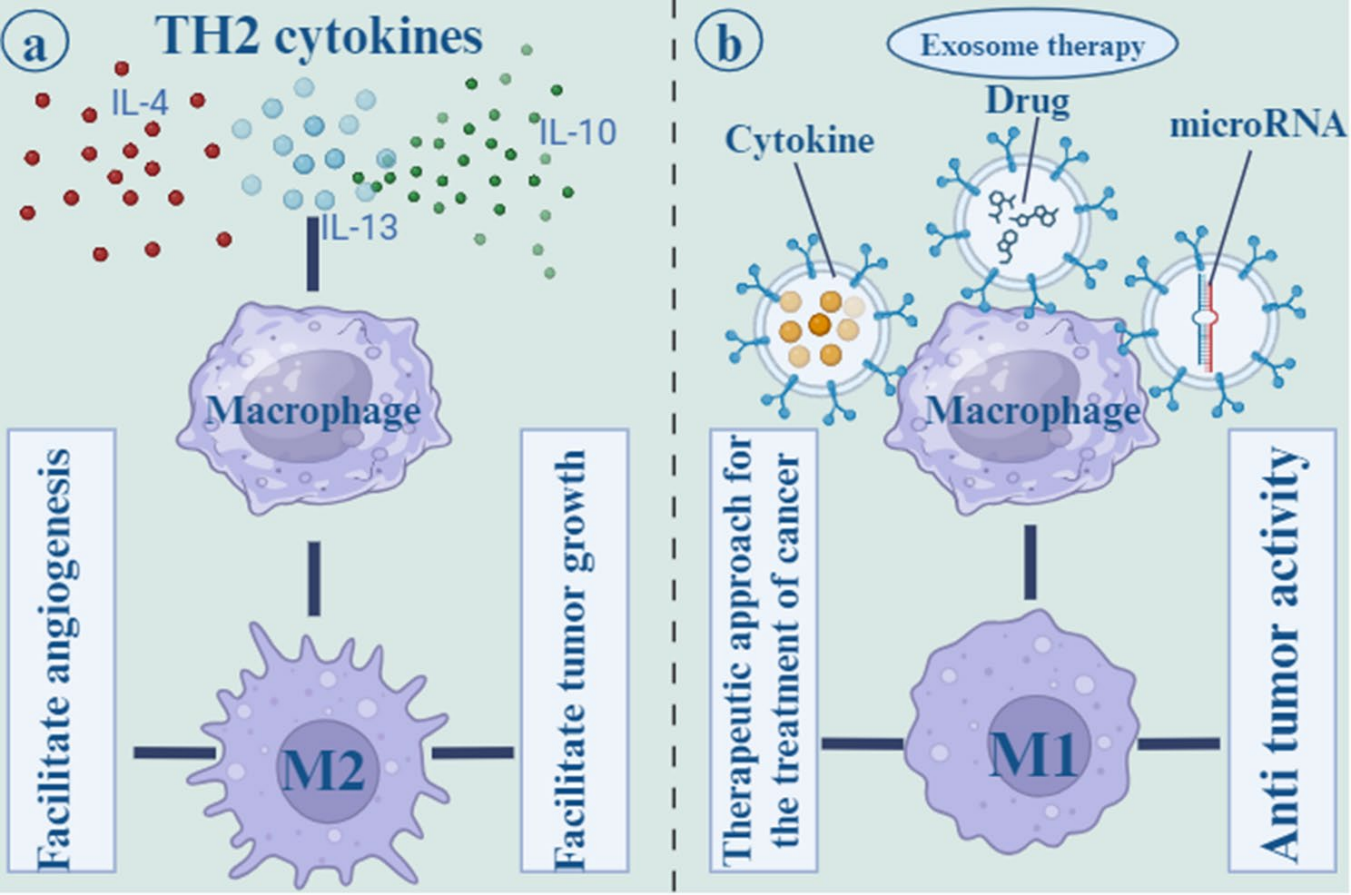
Th2 cytokines, such as IL-4, IL-13, and IL-10 polarize macrophages to the M2 phenotype, which is known to have an anti-inflammatory effect and to play a role in tissue repair and hemostasis. the M2 subtypes, which originate from macrophages in the tumor microenvironment and promote tumor development as well as angiogenesis (**a**). Other methods for polarizing M2 macrophages to the M1 phenotype include the infusion of cytokines and drugs. Additionally, it has been shown that microRNAs are an essential component in the regulation of the polarization of macrophages. For example, mir-155 was able to suppress M2/Th2 responses (**b**)

Suppressing the M2 phenotype or promoting the M1 phenotype in macrophages may be a therapeutic approach for the treatment of cancer. A number of strategies are available to inhibit M2 macrophage attraction and differentiation, including blocking the prostaglandin E2, IL-6, and STAT3 activation loops and preventing tumors from releasing growth factors [111]. Interferon-gamma (IFN-γ) administration or Notch signaling activation are two other approaches for polarizing M2 macrophages to M1 phenotype. Additionally, it has been established that miRNAs play a crucial role in controlling macrophage polarization. For instance, mir-155 inhibited M2/Th2 responses by suppressing CCAAT/enhancer-binding protein beta (C/EBPβ) and interleukin 13 receptor (IL-13R) [113]. MiR-125b by downregulating of IFN regulatory factor 4 proved the potential to activate M1 polarization [114]. Moreover, by transfecting exosomes of MDA-MB-231 cells with miR-130 and miR-33, macrophages were polarized to the M1 phenotype that inhibited tumor growth [115]. MiR-130 was transported via tumor-derived exosomes to M2 macrophages, suppressed tumor cells growth, migration, or invasion [116] (Fig. 6).

### Exosome-coated targeting substance

A new class of tumor-targeting drugs and vector models that target tumor cells within breast cells involves mesenchymal stem cell (MSC) exosomes expressing suicide or tumor-killing genes [117]. In the field of cancer treatment, mesenchymal stem cells are increasingly being sought after because of their ability to differentiate into a wide range of cell types and their powerful immunomodulatory and regenerative potentials [118]. Due to the lack of MHC-II and co-stimulatory ligands, MSCs exhibit poor immunogenicity. They also have good biosafety and are capable of homing to several organs thanks to the production of unique surface molecules [119]. Gene edited MSC-derived exosomes can overexpress a therapeutic RNA or protein to suppress tumors. The treatment of breast cancer can be achieved using MSC exosomes that express suicide or tumor-killing genes [117]. Generally modified MSC exosomes can be effective targeting delivery systems for killing HER2 + tumor cells [120]. O’Brien et al. demonstrated that EVs containing miR-379 suppressed breast tumor through regulation of cyclooxygenase-2 (COX-2) expression [121]. Vakhshiteh et al. harnessed the migration and invasion of MDA-MB-231 cells through exosome secreted by genetically modified dental pulp MSCs (DPSCs) that overexpress miR-34a [122]. Furthermore, LNA-antimir 142-3p was transported effectively by MSCs-Exo and reduced miR-142-3p and miR-150 expression in breast CSC-like cells [123].

Wang et al. targeted HER2 + ve human breast cancer cells via EVs loaded with HChrR6-encoding mRNA and expressing anti-HER2 scFv antibody batch on their surface called “EXO-DEPT”. HChrR6 is a bacterial enzyme that converts 6-chloro-9-nitro-5-oxo-5 H-benzo-(a)-phenoxazine (CNOB) into the cytotoxic drug 9-p amino-6-chloro-5 H-benzo[a]phenoxazine-5-one (MCHB). After treatment, EXO-DEPTs, but not undirected EVs, plus CNOB inhibited almost complete growth of orthotopic BT474 xenografts in vivo [124]. Similarly, Forterre et al. successfully delivered functional HChrR6 mRNA via EVs to HER2 + ve human breast cancer cells. When combined with the prodrug CB1954, they stopped the growth of HER2 + human breast cancer xenografts in athymic mice by activating the prodrug [125]. Overall, these systems are developing, and the successful implementation of them will also contribute to breast cancer treatment.

### Drug delivery carriers

Exosomes can be utilized to transfer conventional clinical chemotherapy drugs to targeted sites, thus reducing their toxicity and improving their enrichment effect [126]. Tran et al. loaded aspirin, as an anti-cancer agent, into exosomes that enhanced drug dissolution and homing targeting effect. Aspirin-loaded exosomes exerts cytotoxicity effects on breast and colorectal cancers [127]. Yu et al. showed that erastin-loaded exosomes labeled with folic acid suppressed the proliferation and migration of MDA-MB-231 cells and induced ferroptosis through the depletion of intracellular glutathione and activation of ROS [88]. In another study, exosomes loaded with paclitaxel (PTX) inhibited tumor progression of MDA-MB-231 cells and were considered an effective drug delivery carrier for BC [128].

Doxorubicin (DOX), a common chemotherapeutic remedy for breast cancer, has been shown to decrease the risk of relapse by up to 8% and death by 6.5%. However, the side effects of congestive heart failure and medicine resistance necessitate switching patients taking doxorubicin to fewer effective therapies. Tian et al. engineered immature DCs (imDCs) expressing Lamp2b, an exosomal membrane protein, fused to αv integrin-specific iRGD peptide. Exosome derived from imDCs loaded with DOX and targeted to αv integrin- positive BC cells. iRGD exosomes efficiently inhibited tumor growth without noticeable toxicity [129]. Li et al. introduced milk exosomes containing DOX to the BC cells with specifically CD44-targeting hyaluronic acid (HA). The HA-mExo-Dox effectively targeted CD44-expressed BC cells and induced apoptosis in vitro [130]. Additionally, Hadla and colleagues confirmed that, in comparison to the free drug, DOX-encapsulated exosomes had less of an impact on cardiac toxicity and other tissues. As a result, the dose of DOX can be enhanced, resulting in a targeted cytotoxic effect on breast cancer cells [126].

## Clinical implications

Achieving an optimal balance between maximal effectiveness and minimum side effects for conventional drugs used to combat tumors is often difficult due to tumor heterogeneity and biological barriers. Many anti-cancer agents in clinical practice have poor bioavailability and little in vivo stability, causing adverse damage to normal cells. Technologies based on exosomes offer exciting approaches to diagnosing and treating BC. Therapeutic agents can be delivered more efficiently using nanoplatforms, which are innovative dosage forms. Besides uploading drugs, nanoplatforms have the ability to target active molecules as well [131].

Nanoplatforms are mainly used to target tumors by enhancing the permeability and retention (EPR) of tumor internals or by interacting with antigens overexpressed on tumor surfaces. A unique carrier property and biosafety make exosomes ideal for developing precisely targeted approaches. In this review, current methods of exosomes in BC treatment are discussed specifically, but there are several challenges to overcome. The production and isolation methods need optimization to obtain sufficient quantities of exosomes with consistent quality and purity. Standardization of isolation, characterization, and quality control methods is crucial for safety and reproducibility. Determining the best administration route is challenging, and different routes need evaluation for effectiveness and safety. Achieving targeted delivery to specific tissues or cells is difficult, but strategies like engineering exosomes with specific ligands are being explored. Loading therapeutic cargo into exosomes is challenging due to limited capacity, and efficient methods are needed for stability and preservation. Exosomes from different cell sources may have varying immunogenicity profiles, so minimizing immune responses is important. Lastly, long-term safety and potential off-target effects or accumulation concerns require careful evaluation [132, 133].

However, through genetic editing, researchers can precisely manipulate specific genes to increase or decrease their expression, thus enhancing anti-tumor exosome production. Exosome surface modification during nanocarrier production can improve exosome recruitment and abundance at tumor locations as well as the recognition of a specific target. Additionally, chemotherapeutics and other therapeutic chemicals like phototherapy and photothermal therapy can be delivered using exosomes as a delivery vehicle. On the other hand, the majority of the current studies involve cell and animal research, and are now in the preclinical stage. It is required to do extensive research on the creation of exosome carriers, how they actually work in the human body, and the management of side effects to establish the combination of both the efficacy and safety of exosome delivery as a way to finally achieve clinical use.

## Data Availability

Not applicable.

## References

[1] Bray F, Ferlay J, Soerjomataram I, Siegel RL, Torre LA, Jemal A (2018). Global cancer statistics 2018: GLOBOCAN estimates of incidence and mortality worldwide for 36 cancers in 185 countries. Cancer J Clin.

[2] Giordano C, La Camera G, Gelsomino L, Barone I, Bonofiglio D, Andò S, Catalano S (2020). The biology of exosomes in breast cancer progression: dissemination, immune evasion and metastatic colonization. Cancers.

[3] Gupta P, Neupane YR, Parvez S, Kohli K (2021). Recent advances in targeted nanotherapeutic approaches for breast cancer management. Nanomedicine.

[4] Harbeck N, Penault-Llorca F, Cortes J, Gnant M, Houssami N, Poortmans P et al. Breast cancer (primer) Nat. Rev Dis Primers. 2019;66. 10.1038/s41572-019-0111-2 31548545

[5] Cheang MC, Martin M, Nielsen TO, Prat A, Voduc D, Rodriguez-Lescure A (2015). Defining breast cancer intrinsic subtypes by quantitative receptor expression. Oncologist.

[6] Wong GL, Abu Jalboush S, Lo H-W (2020). Exosomal MicroRNAs and organotropism in breast cancer metastasis. Cancers.

[7] Amens JN, Bahçecioglu G, Zorlutuna P (2021). Immune System effects on breast Cancer. Cell Mol Bioeng.

[8] Emens LA (2012). Breast cancer immunobiology driving immunotherapy: vaccines and immune checkpoint blockade. Expert Rev Anticancer Ther.

[9] Smyth MJ, Dunn GP, Schreiber RD (2006). Cancer immunosurveillance and immunoediting: the roles of immunity in suppressing tumor development and shaping tumor immunogenicity. Adv Immunol.

[10] Dunn GP, Bruce AT, Ikeda H, Old LJ, Schreiber RD (2002). Cancer immunoediting: from immunosurveillance to tumor escape. Nat Immunol.

[11] Emens LA (2018). Breast Cancer Immunotherapy: facts and HopesBreast Cancer Immunotherapy. Clin Cancer Res.

[12] Edechi CA, Ikeogu N, Uzonna JE, Myal Y (2019). Regulation of immunity in breast cancer. Cancers.

[13] Dunn GP, Old LJ, Schreiber RD (2004). The three Es of cancer immunoediting. Annu Rev Immunol.

[14] Nam GH, Choi Y, Kim GB, Kim S, Kim SA, Kim IS (2020). Emerging prospects of exosomes for cancer treatment: from conventional therapy to immunotherapy. Adv Mater.

[15] Hussen BM, Abdullah KH, Abdullah SR, Majeed NM, Mohamadtahr S, Rasul MF (2023). New insights of miRNA molecular mechanisms in breast cancer brain metastasis and therapeutic targets. Noncoding RNA Res.

[16] Wang H, He D, Wan K, Sheng X, Cheng H, Huang J (2020). In situ multiplex detection of serum exosomal microRNAs using an all-in-one biosensor for breast cancer diagnosis. Analyst.

[17] Yi Y, Wu M, Zeng H, Hu W, Zhao C, Xiong M (2021). Tumor-derived exosomal non-coding RNAs: the emerging mechanisms and potential clinical applications in breast Cancer. Front Oncol.

[18] Zhou Q, Wang J, Zhang H, Sun L, Liu J, Meng L, Li J (2023). Tumor-derived exosomes RNA expression profiling identifies the prognosis, immune characteristics, and treatment in HR+/HER2-breast cancer. Aging.

[19] Zhang J, Shi J, Liu W, Zhang K, Zhao H, Zhang H, Zhang Z (2018). A simple, specific and on-off type MUC1 fluorescence aptasensor based on exosomes for detection of breast cancer. Sens Actuators B.

[20] Kennedy LB, Salama AK (2020). A review of cancer immunotherapy toxicity. Cancer J Clin.

[21] Xu Z, Zeng S, Gong Z, Yan Y (2020). Exosome-based immunotherapy: a promising approach for cancer treatment. Mol Cancer.

[22] Lipson EJ, Forde PM, Hammers H-J, Emens LA, Taube JM, Topalian SL, editors. Antagonists of PD-1 and PD-L1 in cancer treatment. Seminars in oncology. Elsevier; 2015. 10.1053/j.seminoncol.2015.05.013 PMC4555873 26320063

[23] Emens LA, Ascierto PA, Darcy PK, Demaria S, Eggermont AM, Redmond WL (2017). Cancer immunotherapy: opportunities and challenges in the rapidly evolving clinical landscape. Eur J Cancer.

[24] Kamerkar S, LeBleu VS, Sugimoto H, Yang S, Ruivo CF, Melo SA (2017). Exosomes facilitate therapeutic targeting of oncogenic KRAS in pancreatic cancer. Nature.

[25] Théry C, Ostrowski M, Segura E (2009). Membrane vesicles as conveyors of immune responses. Nat Rev Immunol.

[26] Pitt JM, Charrier M, Viaud S, André F, Besse B, Chaput N, Zitvogel L (2014). Dendritic cell–derived exosomes as immunotherapies in the fight against cancer. J Immunol.

[27] Théry C, Zitvogel L, Amigorena S (2002). Exosomes: composition, biogenesis and function. Nat Rev Immunol.

[28] Tkach M, Théry C (2016). Communication by extracellular vesicles: where we are and where we need to go. Cell.

[29] Kalluri R (2016). The biology and function of exosomes in cancer. J Clin Investig.

[30] Aslan C, Maralbashi S, Salari F, Kahroba H, Sigaroodi F, Kazemi T, Kharaziha P (2019). Tumor-derived exosomes: implication in angiogenesis and antiangiogenesis cancer therapy. J Cell Physiol.

[31] Tran T-H, Mattheolabakis G, Aldawsari H, Amiji M (2015). Exosomes as nanocarriers for immunotherapy of cancer and inflammatory diseases. Clin Immunol.

[32] Steinbichler TB, Dudás J, Skvortsov S, Ganswindt U, Riechelmann H (2019). Skvortsova I-I. therapy resistance mediated by exosomes. Mol Cancer.

[33] Pu X, Ma S, Gao Y, Xu T, Chang P, Dong L (2020). Mesenchymal stem cell-derived exosomes: biological function and their therapeutic potential in radiation damage. Cells.

[34] Klinke DJ, Kulkarni YM, Wu Y, Byrne-Hoffman C (2014). Inferring alterations in cell‐to‐cell communication in HER2 + breast cancer using secretome profiling of three cell models. Biotechnol Bioeng.

[35] Ciravolo V, Huber V, Ghedini GC, Venturelli E, Bianchi F, Campiglio M (2012). Potential role of HER2-overexpressing exosomes in countering trastuzumab‐based therapy. J Cell Physiol.

[36] Maas SL, Breakefield XO, Weaver AM (2017). Extracellular vesicles: unique intercellular delivery vehicles. Trends Cell Biol.

[37] Johnstone RM, Adam M, Hammond J, Orr L, Turbide C (1987). Vesicle formation during reticulocyte maturation. Association of plasma membrane activities with released vesicles (exosomes). J Biol Chem.

[38] Ni Z, Zhou S, Li S, Kuang L, Chen H, Luo X (2020). Exosomes: roles and therapeutic potential in osteoarthritis. Bone Res.

[39] Théry C, Amigorena S, Raposo G, Clayton A (2006). Isolation and characterization of exosomes from cell culture supernatants and biological fluids. Curr Protocols cell Biology.

[40] Kumar DN, Chaudhuri A, Aqil F, Dehari D, Munagala R, Singh S (2022). Exosomes as Emerging Drug Delivery and Diagnostic modality for breast Cancer: recent advances in isolation and application. Cancers.

[41] Chen H, Wang L, Zeng X, Schwarz H, Nanda HS, Peng X, Zhou Y. Exosomes, a new star for targeted delivery. Front Cell Dev Biology. 2021:2827. 10.3389/fcell.2021.751079 PMC8531489 34692704

[42] Beach A, Zhang H-G, Ratajczak MZ, Kakar SS (2014). Exosomes: an overview of biogenesis, composition and role in ovarian cancer. J Ovarian Res.

[43] Schmidt O, Teis D (2012). The ESCRT machinery. Curr Biol.

[44] Colombo M, Moita C, Van Niel G, Kowal J, Vigneron J, Benaroch P (2013). Analysis of ESCRT functions in exosome biogenesis, composition and secretion highlights the heterogeneity of extracellular vesicles. J Cell Sci.

[45] Mercier V, Laporte MH, Destaing O, Blot B, Blouin CM, Pernet-Gallay K (2016). ALG-2 interacting protein-X (Alix) is essential for clathrin-independent endocytosis and signaling. Sci Rep.

[46] Février B, Raposo G (2004). Exosomes: endosomal-derived vesicles shipping extracellular messages. Curr Opin Cell Biol.

[47] Trajkovic K, Hsu C, Chiantia S, Rajendran L, Wenzel D, Wieland F (2008). Ceramide triggers budding of exosome vesicles into multivesicular endosomes. Science.

[48] Kalluri R, LeBleu VS (2020). The biology, function, and biomedical applications of exosomes. Science.

[49] Gurunathan S, Kang M-H, Kim J-H (2021). A comprehensive review on factors influences biogenesis, functions, therapeutic and clinical implications of exosomes. Int J Nanomed.

[50] Panigrahi AR, Srinivas L, Panda J (2022). Exosomes: insights and therapeutic applications in cancer. Translational Oncol.

[51] Hong C-S, Sharma P, Yerneni SS, Simms P, Jackson EK, Whiteside TL, Boyiadzis M (2017). Circulating exosomes carrying an immunosuppressive cargo interfere with cellular immunotherapy in acute myeloid leukemia. Sci Rep.

[52] Greening DW, Gopal SK, Xu R, Simpson RJ, Chen W, editors. Exosomes and their roles in immune regulation and cancer. Seminars in cell & developmental biology. Elsevier; 2015. 10.1016/j.semcdb.2015.02.009 25724562

[53] Barros FM, Carneiro F, Machado JC, Melo SA (2018). Exosomes and immune response in cancer: friends or foes?. Front Immunol.

[54] Yang Y, Li C-W, Chan L-C, Wei Y, Hsu J-M, Xia W (2018). Exosomal PD-L1 harbors active defense function to suppress T cell killing of breast cancer cells and promote tumor growth. Cell Res.

[55] Lugini L, Cecchetti S, Huber V, Luciani F, Macchia G, Spadaro F (2012). Immune surveillance properties of human NK cell-derived exosomes. J Immunol.

[56] Huntington ND, Cursons J, Rautela J (2020). The cancer–natural killer cell immunity cycle. Nat Rev Cancer.

[57] Liu J, Ren L, Li S, Li W, Zheng X, Yang Y (2021). The biology, function, and applications of exosomes in cancer. Acta Pharm Sinica B.

[58] Hegmans JP, Gerber PJ, Lambrecht BN, Exosomes. Functional Proteomics: Springer; 2008. p. 97–109. 10.1007/978-1-59745-398-1_7 18592175

[59] Whiteside TL (2013). Immune modulation of T-cell and NK (natural killer) cell activities by TEXs (tumour-derived exosomes). Biochem Soc Trans.

[60] Clayton A, Tabi Z (2005). Exosomes and the MICA-NKG2D system in cancer. Blood Cells Molecules Dis.

[61] Bauernhofer T, Kuss I, Henderson B, Baum AS, Whiteside TL (2003). Preferential apoptosis of CD56dim natural killer cell subset in patients with cancer. Eur J Immunol.

[62] Liu C, Yu S, Zinn K, Wang J, Zhang L, Jia Y (2006). Murine mammary carcinoma exosomes promote tumor growth by suppression of NK cell function. J Immunol.

[63] Wen SW, Sceneay J, Lima LG, Wong CS, Becker M, Krumeich S (2016). The Biodistribution and Immune Suppressive effects of breast Cancer–derived ExosomesExosomes regulate Immune Composition in Metastatic organs. Cancer Res.

[64] Xu HY, Li N, Yao N, Xu XF, Wang HX, Liu XY, Zhang Y (2019). CD8 + T cells stimulated by exosomes derived from RenCa cells mediate specific immune responses through the FasL/Fas signaling pathway and, combined with GM–CSF and IL–12, enhance the anti–renal cortical adenocarcinoma effect. Oncol Rep.

[65] Poggio M, Hu T, Pai C-C, Chu B, Belair CD, Chang A (2019). Suppression of exosomal PD-L1 induces systemic anti-tumor immunity and memory. Cell.

[66] Theodoraki M-N, Yerneni SS, Hoffmann TK, Gooding WE, Whiteside TL (2018). Clinical significance of PD-L1 + exosomes in plasma of Head and Neck Cancer PatientsPD-L1 + exosomes in plasma of HNC patients. Clin Cancer Res.

[67] Wang Y, Goliwas KF, Severino PE, Hough KP, Van Vessem D, Wang H (2020). Mechanical strain induces phenotypic changes in breast cancer cells and promotes immunosuppression in the tumor microenvironment. Lab Invest.

[68] Chatterjee S, Chatterjee A, Jana S, Dey S, Roy H, Das MK (2021). Transforming growth factor beta orchestrates PD-L1 enrichment in tumor-derived exosomes and mediates CD8 T-cell dysfunction regulating early phosphorylation of TCR signalome in breast cancer. Carcinogenesis.

[69] Wada J, Onishi H, Suzuki H, Yamasaki A, Nagai S, Morisaki T, Katano M (2010). Surface-bound TGF-β1 on effusion-derived exosomes participates in maintenance of number and suppressive function of regulatory T-cells in malignant effusions. Anticancer Res.

[70] Yin Y, Cai X, Chen X, Liang H, Zhang Y, Li J (2014). Tumor-secreted miR-214 induces regulatory T cells: a major link between immune evasion and tumor growth. Cell Res.

[71] Kalvala A, Wallet P, Yang L, Wang C, Li H, Nam A (2019). Phenotypic switching of naïve T cells to immune-suppressive Treg-like cells by mutant KRAS. J Clin Med.

[72] Ni C, Fang Q-Q, Chen W-Z, Jiang J-X, Jiang Z, Ye J (2020). Breast cancer-derived exosomes transmit lncRNA SNHG16 to induce CD73 + γδ1 Treg cells. Signal Transduct Target Therapy.

[73] Kumar V, Patel S, Tcyganov E, Gabrilovich DI (2016). The nature of myeloid-derived suppressor cells in the tumor microenvironment. Trends Immunol.

[74] Filipazzi P, Bürdek M, Villa A, Rivoltini L, Huber V, editors. Recent advances on the role of tumor exosomes in immunosuppression and disease progression. Seminars in cancer biology. Elsevier; 2012. 10.1016/j.semcancer.2012.02.005 22369922

[75] Gao Y, Xu H, Li N, Wang H, Ma L, Chen S (2020). Renal cancer-derived exosomes induce tumor immune tolerance by MDSCs-mediated antigen-specific immunosuppression. Cell Communication Signal.

[76] Jiang M, Zhang W, Zhang R, Liu P, Ye Y, Yu W (2020). Cancer exosome-derived miR-9 and miR-181a promote the development of early-stage MDSCs via interfering with SOCS3 and PIAS3 respectively in breast cancer. Oncogene.

[77] Xiang X, Poliakov A, Liu C, Liu Y, Deng Zb, Wang J (2009). Induction of myeloid-derived suppressor cells by tumor exosomes. Int J Cancer.

[78] Fu H, Yang H, Zhang X, Xu W (2016). The emerging roles of exosomes in tumor–stroma interaction. J Cancer Res Clin Oncol.

[79] Yan Z, Sheng Z, Zheng Y, Feng R, Xiao Q, Shi L (2021). Cancer-associated fibroblast-derived exosomal miR-18b promotes breast cancer invasion and metastasis by regulating TCEAL7. Cell Death Dis.

[80] Lee J-K, Park S-R, Jung B-K, Jeon Y-K, Lee Y-S, Kim M-K (2013). Exosomes derived from mesenchymal stem cells suppress angiogenesis by down-regulating VEGF expression in breast cancer cells. PLoS ONE.

[81] Zhu Y, Tao Z, Chen Y, Lin S, Zhu M, Ji W (2022). Exosomal MMP-1 transfers metastasis potential in triple-negative breast cancer through PAR1-mediated EMT. Breast Cancer Res Treat.

[82] Tung KH, Ernstoff MS, Allen C, Shu S (2019). A review of exosomes and their role in the Tumor Microenvironment and Host-Tumor Macroenvironment. J Immunol Sci.

[83] Yu Dd, Wu Y, Shen Hy L, Mm C, Wx Z, Xh (2015). Exosomes in development, metastasis and drug resistance of breast cancer. Cancer Sci.

[84] György B, Hung ME, Breakefield XO, Leonard JN (2015). Therapeutic applications of extracellular vesicles: clinical promise and open questions. Annu Rev Pharmacol Toxicol.

[85] Liang Y, Duan L, Lu J, Xia J (2021). Engineering exosomes for targeted drug delivery. Theranostics.

[86] Huang L, Rong Y, Tang X, Yi K, Qi P, Hou J (2022). Engineered exosomes as an in situ DC-primed vaccine to boost antitumor immunity in breast cancer. Mol Cancer.

[87] Gong C, Tian J, Wang Z, Gao Y, Wu X, Ding X (2019). Functional exosome-mediated co-delivery of doxorubicin and hydrophobically modified microRNA 159 for triple-negative breast cancer therapy. J Nanobiotechnol.

[88] Yu M, Gai C, Li Z, Ding D, Zheng J, Zhang W (2019). Targeted exosome-encapsulated erastin induced ferroptosis in triple negative breast cancer cells. Cancer Sci.

[89] Zhao L, Gu C, Gan Y, Shao L, Chen H, Zhu H (2020). Exosome-mediated siRNA delivery to suppress postoperative breast cancer metastasis. J Controlled Release.

[90] Ohno S-i, Takanashi M, Sudo K, Ueda S, Ishikawa A, Matsuyama N (2013). Systemically injected exosomes targeted to EGFR deliver antitumor microRNA to breast cancer cells. Mol Ther.

[91] Han D, Wang K, Zhang T, Gao G-C, Xu H. Natural killer cell-derived exosome-entrapped paclitaxel can enhance its anti-tumor effect. Eur Rev Med Pharmacol Sci. 2020;24(10). 10.26355/eurrev_202005_21362 32495906

[92] Hashemi ZS, Ghavami M, Kiaie SH, Mohammadi F, Barough MS, Khalili S (2023). Novel delivery of sorafenib by natural killer cell-derived exosomes-enhanced apoptosis in triple-negative breast cancer. Nanomedicine.

[93] Wylie B, Macri C, Mintern JD, Waithman JJC. Dendritic cells and cancer: from biology to therapeutic intervention. 2019;11(4):521. 10.3390/cancers11040521 PMC6521027 30979057

[94] Boer MC, Joosten SA, Ottenhoff THJF. Regulatory T-cells at the interface between human host and pathogens in infectious diseases and vaccination. 2015;6:217. 10.3389/fimmu.2015.00217 PMC4426762 26029205

[95] Admyre C, Johansson SM, Paulie S, Gabrielsson S (2006). Direct exosome stimulation of peripheral humanT cells detected by ELISPOT. Eur J Immunol.

[96] Vincent-Schneider H, Stumptner‐Cuvelette P, Lankar D, Pain S, Raposo G, Benaroch P, Bonnerot C (2002). Exosomes bearing HLA‐DR1 molecules need dendritic cells to efficiently stimulate specific T cells. Int Immunol.

[97] Romagnoli GG, Zelante BB, Toniolo PA, Migliori IK, Barbuto JAM (2015). Dendritic cell-derived exosomes may be a tool for cancer immunotherapy by converting tumor cells into immunogenic targets. Front Immunol.

[98] O’Sullivan TE, Sun JC, Lanier LL (2015). Natural killer cell memory. Immunity.

[99] Di Pace AL, Tumino N, Besi F, Alicata C, Conti LA, Munari E (2020). Characterization of human NK cell-derived exosomes: role of DNAM1 receptor in exosome-mediated cytotoxicity against tumor. Cancers.

[100] Zhu L, Gangadaran P, Kalimuthu S, Oh JM, Baek SH, Jeong SY (2018). Novel alternatives to extracellular vesicle-based immunotherapy–exosome mimetics derived from natural killer cells. Artif Cells Nanomed Biotechnol.

[101] Kaban K, Hinterleitner C, Zhou Y, Salva E, Kantarci AG, Salih HR, Märklin M (2021). Therapeutic silencing of Bcl-2 using Nk cell-derived exosomes as a novel therapeutic approach in breast cancer. Cancers.

[102] Borst J, Ahrends T, Bąbała N, Melief CJ, Kastenmüller W (2018). CD4 + T cell help in cancer immunology and immunotherapy. Nat Rev Immunol.

[103] Wang L, Xie Y, Ahmed KA, Ahmed S, Sami A, Chibbar R (2013). Exosomal pMHC-I complex targets T cell-based vaccine to directly stimulate CTL responses leading to antitumor immunity in transgenic FVBneuN and HLA-A2/HER2 mice and eradicating trastuzumab-resistant tumor in athymic nude mice. Breast Cancer Res Treat.

[104] Hao S, Liu Y, Yuan J, Zhang X, He T, Wu X (2007). Novel exosome-targeted CD4 + T cell vaccine counteracting CD4 + 25 + regulatory T cell-mediated immune suppression and stimulating efficient central memory CD8 + CTL responses. J Immunol.

[105] Xie Y, Wang L, Freywald A, Qureshi M, Chen Y, Xiang J (2013). A novel T cell-based vaccine capable of stimulating long-term functional CTL memory against B16 melanoma via CD40L signaling. Cell Mol Immunol.

[106] Li R, Chibbar R, Xiang J (2018). Novel EXO-T vaccine using polyclonal CD4 + T cells armed with HER2-specific exosomes for HER2-positive breast cancer. OncoTargets Therapy.

[107] Shi X, Cheng Q, Hou T, Han M, Smbatyan G, Lang JE (2020). Genetically engineered cell-derived nanoparticles for targeted breast cancer immunotherapy. Mol Ther.

[108] Cheng Q, Shi X, Zhang Y (2020). Reprogramming exosomes for Immunotherapy.

[109] Cao Y, Rodgers DT, Du J, Ahmad I, Hampton EN, Ma JS (2016). Design of switchable chimeric antigen receptor T cells targeting breast cancer. Angew Chem Int Ed.

[110] Yang P, Cao X, Cai H, Feng P, Chen X, Zhu Y (2021). The exosomes derived from CAR-T cell efficiently target mesothelin and reduce triple-negative breast cancer growth. Cell Immunol.

[111] Heusinkveld M, van Der Burg SH (2011). Identification and manipulation of tumor associated macrophages in human cancers. J Translational Med.

[112] Ham S, Lima LG, Chai EPZ, Muller A, Lobb RJ, Krumeich S (2018). Breast cancer-derived exosomes alter macrophage polarization via gp130/STAT3 signaling. Front Immunol.

[113] O’Connell RM, Taganov KD, Boldin MP, Cheng G, Baltimore D. MicroRNA-155 is induced during the macrophage inflammatory response. Proceedings of the National Academy of Sciences. 2007;104(5):1604-9. 10.1073/pnas.0610731104 PMC1780072 17242365

[114] Chaudhuri AA, So AY-L, Sinha N, Gibson WS, Taganov KD, O’Connell RM, Baltimore D (2011). MicroRNA-125b potentiates macrophage activation. J Immunol.

[115] Moradi-Chaleshtori M, Bandehpour M, Soudi S, Mohammadi-Yeganeh S, Hashemi SM (2021). In vitro and in vivo evaluation of anti-tumoral effect of M1 phenotype induction in macrophages by miR-130 and miR-33 containing exosomes. Cancer Immunol Immunother.

[116] Moradi-Chaleshtori M, Shojaei S, Mohammadi-Yeganeh S, Hashemi SM (2021). Transfer of miRNA in tumor-derived exosomes suppresses breast tumor cell invasion and migration by inducing M1 polarization in macrophages. Life Sci.

[117] Altanerova U, Jakubechova J, Benejova K, Priscakova P, Pesta M, Pitule P (2019). Prodrug suicide gene therapy for cancer targeted intracellular by mesenchymal stem cell exosomes. Int J Cancer.

[118] Lee J, Henderson K, Massidda MW, Armenta-Ochoa M, Im BG, Veith A (2021). Mechanobiological conditioning of mesenchymal stem cells for enhanced vascular regeneration. Nat Biomedical Eng.

[119] Li H, Jin Y, Zhao Y, Li W, He Z, Zhang Q (2021). Targeted cell therapy for partial-thickness cartilage defects using membrane modified mesenchymal stem cells by transglutaminase 2. Biomaterials.

[120] Gomari H, Forouzandeh Moghadam M, Soleimani M. Targeted cancer therapy using engineered exosome as a natural drug delivery vehicle. OncoTargets Therapy. 2018:5753–62. 10.2147/OTT.S173110 PMC6140699 30254468

[121] O’brien K, Khan S, Gilligan K, Zafar H, Lalor P, Glynn C (2018). Employing mesenchymal stem cells to support tumor-targeted delivery of extracellular vesicle (EV)-encapsulated microRNA-379. Oncogene.

[122] Vakhshiteh F, Rahmani S, Ostad SN, Madjd Z, Dinarvand R, Atyabi F (2021). Exosomes derived from miR-34a-overexpressing mesenchymal stem cells inhibit in vitro tumor growth: a new approach for drug delivery. Life Sci.

[123] Naseri Z, Oskuee RK, Forouzandeh-Moghadam M, Jaafari MR (2020). Delivery of LNA-antimiR-142-3p by mesenchymal stem cells-derived exosomes to breast cancer stem cells reduces tumorigenicity. Stem cell Reviews Rep.

[124] Wang J-H, Forterre AV, Zhao J, Frimannsson DO, Delcayre A, Antes TJ (2018). Anti-HER2 scfv-directed extracellular vesicle-mediated mRNA-based gene delivery inhibits growth of HER2-positive human breast tumor xenografts by prodrug activation. Mol Cancer Ther.

[125] Forterre AV, Wang J-H, Delcayre A, Kim K, Green C, Pegram MD (2020). Extracellular vesicle–mediated in Vitro transcribed mRNA delivery for treatment of HER2 + breast Cancer xenografts in mice by Prodrug CB1954 without General ToxicitySide Effect–Free GDEPT in mice by CB1954 (Tretazicar). Mol Cancer Ther.

[126] Hadla M, Palazzolo S, Corona G, Caligiuri I, Canzonieri V, Toffoli G, Rizzolio F (2016). Exosomes increase the therapeutic index of doxorubicin in breast and ovarian cancer mouse models. Nanomedicine.

[127] Tran PH, Wang T, Yin W, Tran TT, Barua HT, Zhang Y (2019). Development of a nanoamorphous exosomal delivery system as an effective biological platform for improved encapsulation of hydrophobic drugs. Int J Pharm.

[128] Kalimuthu S, Gangadaran P, Rajendran RL, Zhu L, Oh JM, Lee HW (2018). A new approach for loading anticancer drugs into mesenchymal stem cell-derived exosome mimetics for cancer therapy. Front Pharmacol.

[129] Tian Y, Li S, Song J, Ji T, Zhu M, Anderson GJ (2014). A doxorubicin delivery platform using engineered natural membrane vesicle exosomes for targeted tumor therapy. Biomaterials.

[130] Li D, Yao S, Zhou Z, Shi J, Huang Z, Wu Z (2020). Hyaluronan decoration of milk exosomes directs tumor-specific delivery of doxorubicin. Carbohydr Res.

[131] Jung I, Shin S, Baek M-C, Yea K. Modification of immune cell-derived exosomes for enhanced cancer immunotherapy: current advances and therapeutic applications. Exp Mol Med. 2024:1–13. 10.1038/s12276-023-01132-8 PMC10834411 38172594

[132] Yamashita T, Takahashi Y, Takakura Y (2018). Possibility of exosome-based therapeutics and challenges in production of exosomes eligible for therapeutic application. Biol Pharm Bull.

[133] Willis GR, Kourembanas S, Mitsialis SA (2017). Toward exosome-based therapeutics: isolation, heterogeneity, and fit-for-purpose potency. Front Cardiovasc Med.

